# Robust adaptive filtering algorithms based on (inverse)hyperbolic sine function

**DOI:** 10.1371/journal.pone.0258155

**Published:** 2021-10-11

**Authors:** Sihai Guan, Qing Cheng, Yong Zhao, Bharat Biswal

**Affiliations:** 1 College of Electronic and Information, Southwest Minzu University, Chengdu, China; 2 Key Laboratory of Electronic and Information Engineering, State Ethnic Affairs Commission, Chengdu, China; 3 Sichuan Vocational College of Finance and Economics, Chengdu, China; 4 School of Mechanical and Power Engineering, Henan Polytechnic University, Jiaozuo, China; 5 The Clinical Hospital of Chengdu Brain Science Institute, MOE Key Laboratory for Neuroinformation, Center for Information in Medicine, School of Life Science and Technology, University of Electronic Science and Technology of China, Chengdu, China; 6 Department of Biomedical Engineering, New Jersey Institute of Technology (NJIT), Newark, NJ, United States of America; University of Bradford, UNITED KINGDOM

## Abstract

Recently, adaptive filtering algorithms were designed using hyperbolic functions, such as hyperbolic cosine and tangent function. However, most of those algorithms have few parameters that need to be set, and the adaptive estimation accuracy and convergence performance can be improved further. More importantly, the hyperbolic sine function has not been discussed. In this paper, a family of adaptive filtering algorithms is proposed using hyperbolic sine function (HSF) and inverse hyperbolic sine function (IHSF) function. Specifically, development of a robust adaptive filtering algorithm based on HSF, and extend the HSF algorithm to another novel adaptive filtering algorithm based on IHSF; then continue to analyze the computational complexity for HSF and IHSF; finally, validation of the analyses and superiority of the proposed algorithm via simulations. The HSF and IHSF algorithms can attain superior steady-state performance and stronger robustness in impulsive interference than several existing algorithms for different system identification scenarios, under Gaussian noise and impulsive interference, demonstrate the superior performance achieved by HSF and IHSF over existing adaptive filtering algorithms with different hyperbolic functions.

## 1. Introduction

Adaptive filter (AF) algorithms are frequently employed in linear systems [1–3], nonlinear systems [4], and distributed network systems [5] and have been used in many fields, including biomedical engineering [6,7]. Among adaptive filter algorithms, the least mean square (LMS) algorithm has probably become the most popular adaptive filtering algorithm for its simple configuration, low computational complexity, sufficient tracking capability, and easiness of implementation [2,3,5,7–13]. However, in actual engineering, non-Gaussian distribution measurement noise with a heavy-tailed pdf, e.g., a Laplace or α-stable noise, is everywhere [8,12,14]. However, those measurement noises significantly affect the adaptive estimation accuracy of estimation, and most of the adaptive estimation algorithms are highly susceptible to impulsive interference [8,12]. In the presence of non-Gaussian noise, adaptive filters are required to be less sensitive to large noises and be more sensitive to small noises [15]. If the measurement noise involves impulse interference, the adaptive filtering algorithm based on the mean square error criterion may severely reduce the convergence performance and even cause divergence problems [16–18]. In such cases, the commonly used cost functions include high order error power (HOEP) conditions [19–24], the mixed-norm cost function [12,25], the logarithmic and exponential cost functions [15,26], the correntropy cost function [27], and the kernel risk-sensitive cost function [28]. The most common HOEP algorithm is the least mean absolute-third [20,21] and least mean fourth (LMF) [22,24,29,30] algorithms. The LMF algorithm outperforms the LMS algorithm and achieves a better trade-off between the LMS’s transient and steady-state performances [22,24,29,30]; the computational complexity of LMF is very high. Besides, to fully take advantage of both LMS and LMF, Lim and colleagues proposed a combined LMS/F algorithm [31], and its simplicity and stability developed in [32,33]. Besides, based on the mean absolute error (MAE) [34], the sign-error-type algorithm generally exhibits a slow convergence rate in practice. Based on information-theoretic learning [35], Chen and colleagues proposed the generalized maximum correntropy criterion (GMCC) based adaptive filtering robust under non-Gaussian/impulsive noise [27]. However, the correntropy’s performance surface is sharp around the optimal solution but flat far away from the optimal solution [28]. In robust adaptive filters, the robust LMS algorithm [36] utilizes a linear combination of bounded hyperbolic tangent basis functions to approximate the optimal score function. The correntropy or the logarithmic hyperbolic function is considered a candidate for the cost function in the switching algorithm for super-Gaussian noises due to their robustness [37]. In an endeavor to achieve lower steady-state misalignment, a generalized hyperbolic secant function as a robust norm and derive the generalized hyperbolic secant adaptive filter was proposed [38]. To address both Gaussian and non-Gaussian noises with a uniform expression, Liu and colleagues [39] proposed a novel HTCC algorithm by combining nonlinear function and mapping mode. And hyperbolic tangent function was also constructed in a drought disaster losses model, which was used to analyze the characteristics of a drought disaster losses curve with multiple inflection points and nonlinearity [40]. In addition, to reduce the interference of impulse noise on the traditional spline adaptive filter algorithm for identifying Wiener-type non-systems, an arctangent function that is insensitive to large outliers is used to construct a cost function, so that the spline adaptive filter is robustness to impulse noise [41]. Since it is difficult to obtain an analytical result of the expectation of the hyperbolic tangent function under the Gaussian assumption, the hyperbolic tangent-based robust filter and its performance analysis are not considered in both robust LMS and the switching algorithm. Wang and colleagues proposed a logarithmic hyperbolic cosine adaptive filter (LHCAF) [42] by using only the logarithmic hyperbolic cosine based cost function. LHCAF was shown to provide better convergence performance compared to the well-known GMCC [27], it may not provide optimal performance as it does not take into account the sparse nature of the system. Then, a sparsity-aware zero attraction LHCAF and a reweighted zero attraction LHCAF were proposed [43]. Besides, Liang and colleagues [44] developed a recursive constrained least lncosh adaptive filtering algorithm to suppress impulsive interference. Also for dealing with non-Gaussian distribution noise signal, an adaptive filtering algorithm, namely least lncosh (Llncosh) algorithm was proposed, and VLlncosh scheme PLlncosh algorithm were also extend [45]. Inspired by the hyperbolic secant cost function, Lu and colleague [46] improved performance of the adaptive filtering algorithm in white Gaussian noise and uniform noise environments. Recently, Tao and colleagues [47] proposed a constrained least lncosh adaptive filtering algorithm under non-Gaussian noise environment.

Although a class of adaptive filtering algorithms was designed using the hyperbolic function, most of those algorithms have few parameters that need to be set. The estimation accuracy and convergence performance of those adaptive algorithms can be improved further. More importantly, the hyperbolic sine function has not yet been discussed. This paper proposed a family of hyperbolic sine functions, including joint hyperbolic sine function (HSF) and inverse hyperbolic sine function (IHSF), as cost functions. Compared with several existing adaptive filtering algorithms, superior steady-state performance and stronger robustness can be attained. Extensive simulation studies for different system identification scenarios under Gaussian and non-Gaussian disturbances demonstrate the superior performance achieved by HSF and IHSF over existing robust adaptive filtering algorithms.

This paper further studies and improves how to design an adaptive filtering algorithm using hyperbolic sine function as the cost function. To summarize, the main contributions of this paper are: (1) development of a robust adaptive filtering algorithm based on hyperbolic sine function (HSF); (2) then extend the HSF algorithm to another novel adaptive filtering algorithm based on inverse hyperbolic sine function (IHSF); (3) analyses of the computational complexity for the HSF and IHSF algorithms; (4) validation of the analyses and superiority of the proposed algorithm via simulations. Moreover, the first step in this paper’s schematic diagram is to list current research results, then point out which ones can be further studied. The rest of this paper is arranged as follows. In Section 2, the HSF and IHSF algorithms are derived based on the hyperbolic sine function and the inverse hyperbolic sine function. In Section 3, the computational complexity of HSF and IHSF algorithms is analyzed. Section 4 provides several experiments simulation results. Finally, Section 5 gives a conclusion. Note: Bold type refers to vectors, [∙]^T^ denotes for the transpose, and [∙]^−1^ denotes the inverse operation.

## 2. Hyperbolic sine function

One of the cost functions is given by

$$J_{HSF}(e(n))=E[sinh(e^{2}(n))]$$ (1)

where sinh(∙) is the hyperbolic sine function which is expressed as

$$sinh(e^{2}(n))=\frac{exp(e^{2}(n))-exp(-e^{2}(n))}{2}$$ (2)

And then, the score function can be given by

$$\frac{\partial J(e(n))}{\partial e(n)}=\frac{\partial }{\partial e(n)}\left(sinh(e^{2}(n))\right)=2cosh(e^{2}(n))e(n)$$ (3)

where cosh(∙) is the hyperbolic cosine function which is expressed as

$$cosh(e^{2}(n))=\frac{exp(e^{2}(n))+exp(-e^{2}(n))}{2}$$ (4)

Another cost function is given by

$$J_{IHSF}(e(n))=E[{sinh}^{-1}(e^{2}(n))]$$ (5)

The score function can be given by

$$\frac{\partial J(e(n))}{\partial e(n)}=\frac{\partial }{\partial e(n)}\left({sinh}^{-1}(e^{2}(n))\right)=2\frac{1}{\sqrt{1+e^{2}(n)}}e(n)$$ (6)

According to the definition of the hyperbolic sine function, strongly convex at *e*(*n*)∈(−∞,+∞) are presented as follows

### Proof of convex function

The second derivative of *J*_*HSF*_(*e*(*n*)) concerning *e*(*n*) is

$$\begin{array}{l} \frac{\partial^{2}J(e(n))}{\partial^{2}e(n)}\\ =\frac{\partial^{2}}{\partial^{2}e(n)}(sinh(e^{2}(n)))\\ =\frac{\partial }{\partial e(n)}(2cosh(e^{2}(n))e(n))\\ =2cosh(e^{2}(n))+e(n)\frac{\partial }{\partial e(n)}(2cosh(e^{2}(n)))\\ =2cosh(e^{2}(n))+4e^{2}(n)sinh(e^{2}(n))\\ =(2e^{2}(n)+1)exp(e^{2}(n))-(2e^{2}(n)-1)exp(-e^{2}(n)) \end{array}$$ (7)

Based on Eq (7)

$$\left\{ \begin{array}{c} \underset{e(n)\to 0}{\lim}\frac{\partial^{2}J(e(n))}{\partial^{2}e(n)}=2\\ \underset{e(n)\to \infty }{\lim}\frac{\partial^{2}J(e(n))}{\partial^{2}e(n)}=\infty \end{array}\right.$$ (8)

So, $\frac{\partial^{2}J(e(n))}{\partial^{2}e(n)}>2$, i.e., the hyperbolic sine function is strongly convex at *e*(*n*)∈(−∞,+∞).

### Proof of convex function

The second derivative of *J*_*HSF*_(*e*(*n*)) concerning *e*(*n*) is

$$\begin{array}{l} \frac{\partial^{2}J(e(n))}{\partial^{2}e(n)}\\ =\frac{\partial^{2}}{\partial^{2}e(n)}({sinh}^{-1}(e^{2}(n)))\\ =\frac{\partial }{\partial e(n)}\left(2\frac{1}{\sqrt{1+e^{2}(n)}}e(n)\right)\\ =2\frac{1}{\sqrt{1+e^{2}(n)}}+e(n)\frac{\partial }{\partial e(n)}\left(2\frac{1}{\sqrt{1+e^{2}(n)}}\right)\\ =\frac{2}{\sqrt{1+e^{2}(n)}}+2e(n)\left(-\frac{2e(n)}{{2(1+e^{2}(n))}^{\frac{3}{2}}}\right)\\ =\frac{2}{{\left(1+e^{2}(n)\right)}^{\frac{3}{2}}}>0 \end{array}$$ (9)

So, the inverse hyperbolic sine function is strongly convex at *e*(*n*)∈(−∞,+∞).

Overall, this means that the adaptive filtering algorithm designed based on these two cost functions can be the better estimation result.

## 3. Proposed algorithm based on the hyperbolic sine function

We consider a system identification problem in which the desired signal is generated by

$$d(n)=W_{O}^{T}X(n)+\rho (n)$$ (10)

where *ρ*(*n*) is a stationary additive noise with zero mean and variance of $\sigma_{\rho }^{2}$. Also, {*ρ*(*n*)} is a stationary sequence of independent zero-mean random variables with a finite variance $\sigma_{\rho }^{2}$. Furthermore, it is zero odd-order moments and is assumed to be uncorrelated with any other signal. **W**_*O*_*ϵR*^*L*×1^ is the unknown *L*-dimensional system parameter vector. **X**(*n*) is also stationary with zero-mean, a variance of $\sigma_{x}^{2}$, and **X**(*n*) is Gaussian with a positive-definite autocorrelation matrix **R**_***XX***_ = E[**X**(*n*)**X**^T^(*n*)].

The system estimation error signal can be expressed as

e(n)=d(n)−y(n) (11)

The corresponding filter output is

$$y(n)=W^{T}(n)X(n)$$ (12)

Assuming

$$V(n)=W(n)-W_{O}$$ (13)

where **W**_*O*_ denotes the weight vector of the unknown system with the length of *L*.

So

$$e(n)=W_{O}^{T}X(n)+\rho (n)-W^{T}(n)X(n)={-V}^{T}(n)X(n)+\rho (n)$$ (14)

### 3.1 Least hyperbolic sine function

Based on the error signal *e*(*n*), various error optimization criteria have been developed in search of an optimal solution. For tractability, using the instantaneous error instead of the expectation in Eq (1) as usual, so in this work, one of the cost function used for obtaining the proposed adaptive filtering algorithm is given by

$$W(n)=argminJ(W(n))=argmin[sinh(e^{2}(n))]$$ (15)

The gradient term for the optimization algorithm is given as:

$$\frac{\partial J(W(n))}{\partial W(n)}=\frac{\partial }{\partial W(n)}(sinh(e^{2}(n)))=-2cosh(e^{2}(n))e(n)X(n)$$ (16)

where cosh(∙) is the hyperbolic cosine function which is expressed as

$$cosh(e^{2}(n))=\frac{exp(e^{2}(n))+exp(-e^{2}(n))}{2}$$ (17)

The iterative scheme is given by

$$W(n+1)=W(n)-\mu \frac{\partial J(W(n))}{\partial W(n)}=W(n)+2\mu cosh(e^{2}(n))e(n)X(n)$$ (18)

where *μ* is the step-size (or learning-ratio).

### 3.2 Least inverse hyperbolic sine function

Based on the error signal *e*(*n*), various error optimization criteria have been developed in search of an optimal solution. For tractability, using the instantaneous error instead of the expectation in Eq (5) as usual, in this work, another cost function used for obtaining the proposed adaptive filtering algorithm is given by

$$W(n)=argminJ(W(n))=argmin[{sinh}^{-1}(e^{2}(n))]$$ (19)

The gradient term for the optimization algorithm is given as:

$$\frac{\partial J(W(n))}{\partial W(n)}=\frac{\partial }{\partial W(n)}({sinh}^{-1}(e^{2}(n)))=-2e(n)X(n)$$ (20)

The iterative scheme is given by

$$W(n+1)=W(n)-\mu \frac{\partial J(W(n))}{\partial W(n)}=W(n)+2\mu \frac{1}{\sqrt{1+e^{2}(n)}}e(n)X(n)$$ (21)

where *μ* is the step-size (or learning-ratio).

### 3.3 Computational complexity

Computational complexity is an important property that influences an adaptive filtering algorithm’s performance. The adaptive filtering algorithm’s computational complexity is called the number of arithmetic operations per iteration of the weight vector or coefficient vector. That is the number of multiplications, additions, and et.al. The multiplication operation’s time-consuming operation is far greater than the addition operation’s time-consuming operation, so the multiplication operation occupies a large proportion of the adaptive filtering algorithm’s computational complexity. Therefore, each implementation’s total number of multiplications provided reasonably accurate comparative estimates of their overall complexity. In our proposed HSF and IHSF algorithms, there is no updated step-size formula compared to the VSS-LHCAF [42] and VSS-Llncosh [45] algorithms, meaning that the computational complexity of HSF and IHSF algorithms is smaller than that of the VSS-LHCAF [42] and VSS-Llncosh[45] algorithms. There is also no normalized required to compute, as is the case with the adaptive filtering algorithm with arctangent cost [48]. The HSF and IHSF algorithms also reduce the number of parameters that need to be set. Besides, through comparison, it is also found that there is no *λ* or *λ*(*n*) in HSF and IHSF; that is, there are no artificially set parameters in the algorithms of HSF and IHSF, which further reduces the complexity of the algorithm. For convenience, the weight vector iterative scheme of the VSS-LHCAF [42], VSS-Llncosh [45], adaptive filter with secant cost [46], with arctangent cost [48], the proposed HSF and IHSF algorithms are listed in **Table 1**.

**Table 1:** The weight vector iterative scheme of adaptive filters by using the hyperbolic function.

Algorithm	the weight vector iterative scheme
VSS-LHCAF [42]	**W**(*n*+1) = **W**(*n*)+*μ*(*n*)tanh(*λ*(*n*)*e*(*n*))**X**(*n*)
VSS-Llncosh [45]	**W**(*n*+1) = **W**(*n*)+*μ*(*n*)tanh(*λe*(*n*))**X**(*n*)
with secant cost [46]	$W(n+1)=W(n)+\mu \left(\frac{2sinh(e^{2}(n))}{{\left(cosh(e^{2}(n))\right)}^{2}}+\lambda \right)e(n)X(n)$
with arctangent cost [48]	$W(n+1)=W(n)+\mu \frac{\lambda {\left\|X(n)\right\|}^{2}}{{\left\|X(n)\right\|}^{4}+\lambda^{2}e^{4}(n)}e(n)X(n)$
Proposed HSF	**W**(*n*+1) = **W**(*n*)+2*μ*cosh(*e*^2^(*n*))*e*(*n*)**X**(*n*)
Proposed IHSF	$W(n+1)=W(n)+2\mu \frac{1}{\sqrt{1+e^{2}(n)}}e(n)X(n)$

## 4. Simulation results

This section presents the simulation experiments in system identification using various distributions noise of an unknown system, then using those experiments simulation results to see how robust the proposed algorithms are and illustrate the proposed algorithms’ adaptive estimation accuracy. The misadjustment performance of the VSS-LHCAF [42], VSS-Llncosh [45], adaptive filter with secant cost [46], with arctangent cost [48], HSF, and IHSF algorithms are compared. Besides, the input signal **X**_**UC**_(*n*) is Gaussian white noise with zero mean and $\sigma_{x}^{2}=1$. The correlated input signal **X**_**C**_(*n*) is calculated by using **X**_**C**_(*n*) = 0.95**X**_**C**_(*n*)+**X**_**UC**_(*n*). In all of our experiments, the coefficient vectors are initialized as zero vectors. Impulsive distribution noise and Gaussian noise as a measurement noise are used in those experiments. $MSE(n)=10log10({\left\|d(n)-y(n)\right\|}_{2}^{2})$ is used to measure the steady-state performance of the adaptive fitering algorithm. The results are obtained via Monte Carlo simulation using 10 independent run sets and an iteration number of 6000.

**Fig 1:**
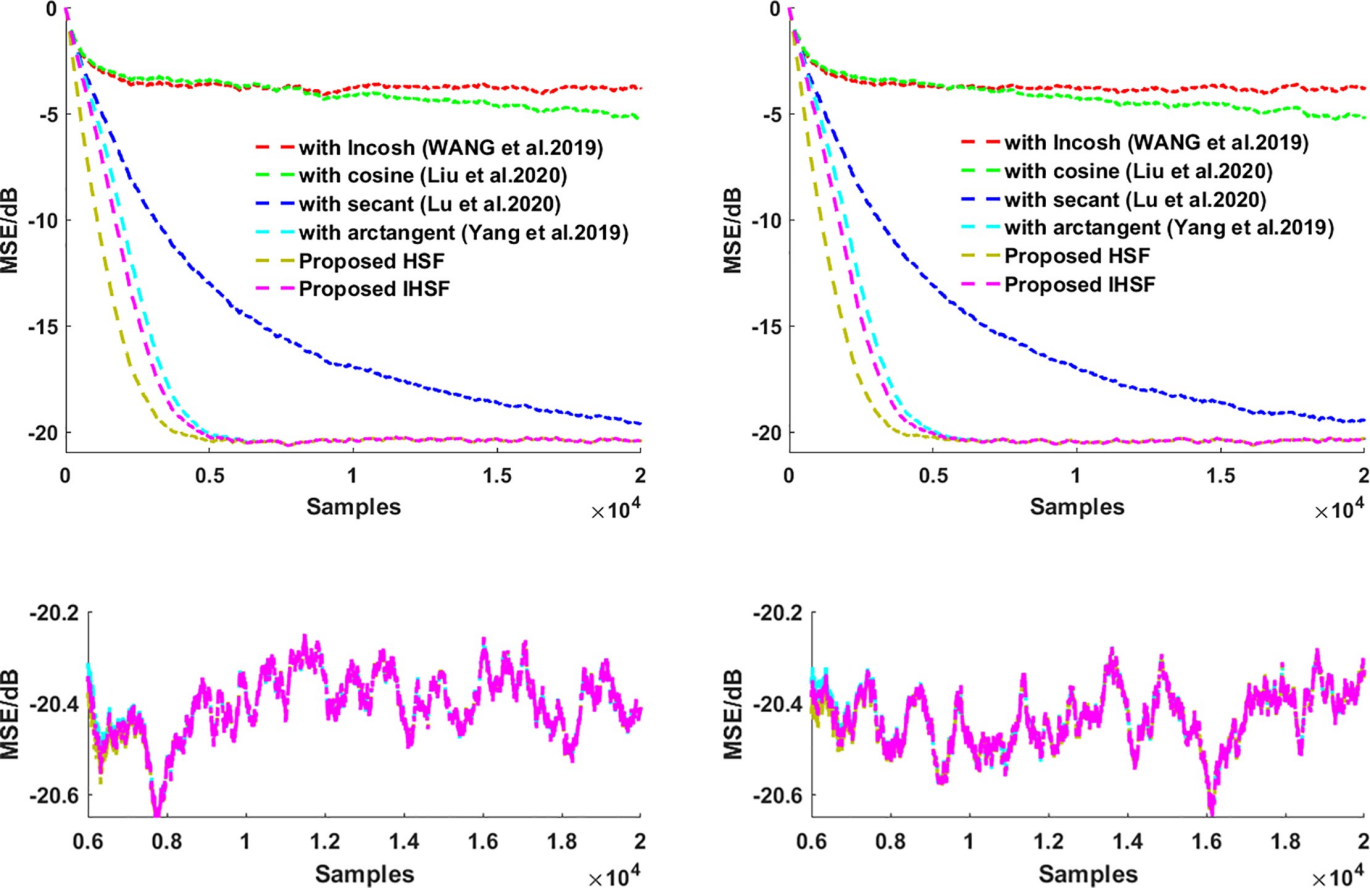
MSEs for the adaptive filtering algorithms by using hyperbolic functions. (left) with the uncorrelated input signal **X**_**UC**_(*n*); (right) with the correlated input signal **X**_**C**_(*n*).

**Fig 2:**
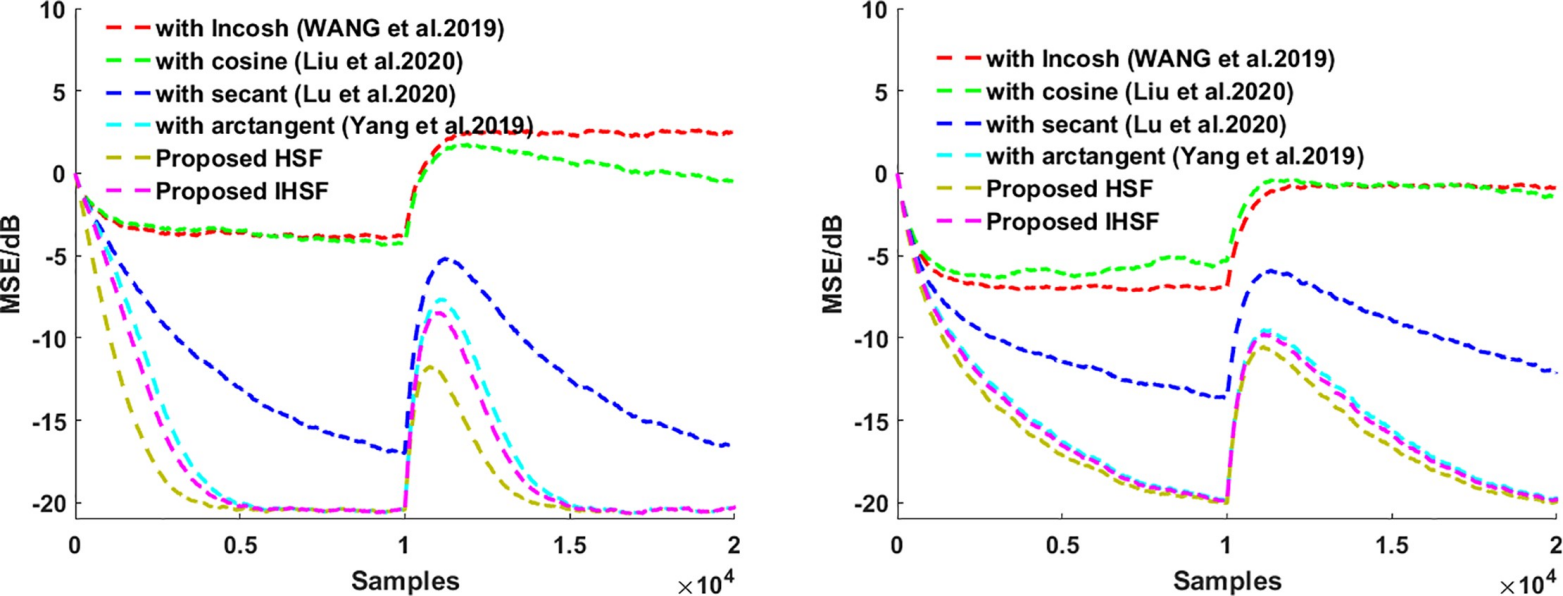
MSEs for the adaptive filtering algorithms by using hyperbolic functions. (left) with the uncorrelated input signal **X**_**UC**_(*n*); (right) with the correlated input signal **X**_**C**_(*n*).

**Fig 3:**
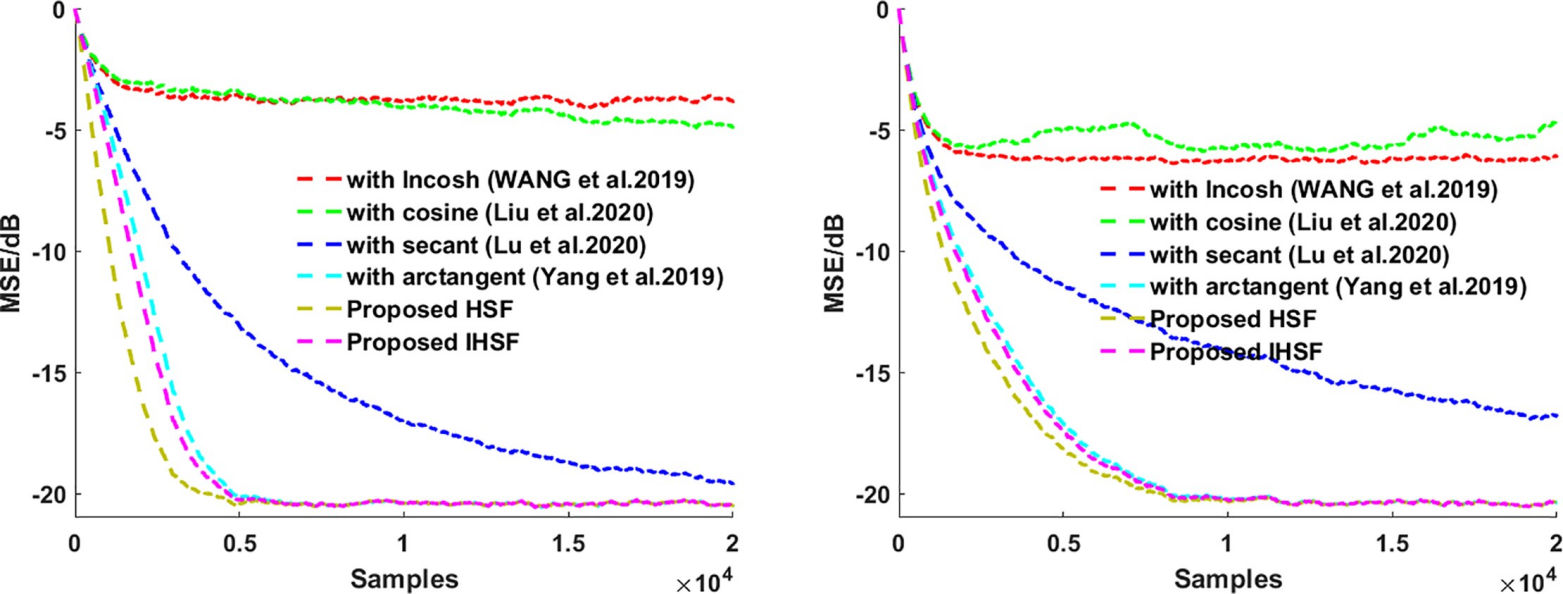
MSEs for the adaptive filtering algorithms by using hyperbolic functions. (left) with the uncorrelated input signal **X**_**UC**_(*n*); (right) with the correlated input signal **X**_**C**_(*n*).

**Fig 4:**
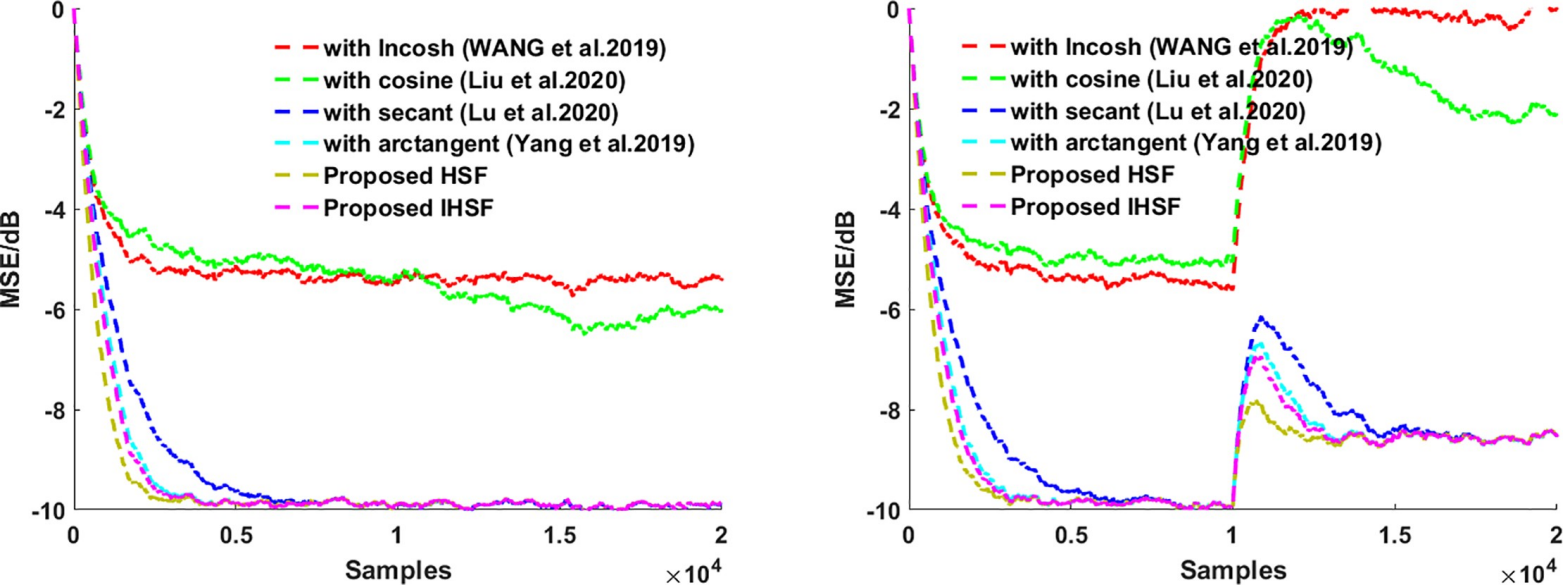
MSEs for the adaptive filtering algorithms by using hyperbolic functions. (left) with the uncorrelated input signal **X**_**UC**_(*n*); (right) with the correlated input signal **X**_**C**_(*n*).

Then using **Figs 1**–**4** to see how the proposed algorithms outperform the VSS-LHCAF, VSS-Llncosh, adaptive filter with secant cost, with arctangent cost algorithms with the uncorrelated input signal **X**_**UC**_(*n*) and the correlated input signal **X**_**C**_(*n*) for Gaussian noise and impulsive interference when the correlated and uncorrelated input signal, respectively. **Figs 1**–**4** shows that the proposed algorithms’ convergence rate is faster than that of the VSS-LHCAF, VSS-Llncosh, adaptive filter with secant cost, with arctangent cost algorithms. Moreover, the proposed HSF and IHSF algorithms have a minor steady-state misalignment (i.e., steady-state estimation error equal to SNR). Compared with the VSS-LHCAF, VSS-Llncosh, adaptive filter with secant cost, with arctangent cost algorithms, the proposed algorithms are more robust to Gaussian noise, impulsive interference, and various types of input signals.

### (1). Effect of impulsive interference for a time-invariant system

The system noise contains Gaussian white noise and impulsive interference with SNR = 20dB. The length of the unknown coefficient vector **W**_***O***_ = [0.6,−0.4,0.25,−0.15,0.1,−0.05,0.001]^T^ is *L* = 7. Parameters are set as the VSS-LHCAF {*μ* = 0.0005}, VSS-Llncosh {*A*_0_ = 0.1,h = 0.1,λ_0_ = 5,*μ* = 0.0005}, adaptive filter with secant cost {λ_0_ = 0.1,*μ* = 0.0005}, with arctangent cost {*μ* = 0.0005}, and the proposed HSF and IHSF algorithms {*μ* = 0.0005}.

### (2). Effect of impulsive interference for a time-varying system

The system noise contains Gaussian white noise and impulsive interference with SNR = 20dB. The length of the unknown coefficient vector *L* = 7, when iteration number smaller than 3000: **W**_***O***_ = [0.6,−0.4,0.25,−0.15,0.1,−0.05,0.001]^T^, when iteration number larger than 3000: **W**_***O***_ = 2×[0.6,−0.4,0.25,−0.15,0.1,−0.05,0.001]^T^. Parameters are set as the VSS-LHCAF {*μ* = 0.0005}, VSS-Llncosh {*α*_0_ = 0.1,h = 0.1,λ_0_ = 5,*μ* = 0.0005}, adaptive filter with secant cost {λ_0_ = 0.1,*μ* = 0.0005}, with arctangent cost {*μ* = 0.0005}, and the proposed HSF and IHSF algorithms {*μ* = 0.0005}.

### (3). Effect of the input signal

The system noise contains Gaussian white noise with SNR = 20dB. The length of the unknown coefficient vector **W**_***O***_ = [0.6,−0.4,0.25,−0.15,0.1,−0.05,0.001]^T^ is *L* = 7. Parameters are set as the VSS-LHCAF, VSS-Llncosh, adaptive filter with secant cost, arctangent cost, and the proposed HSF and IHSF algorithms.

### (4). Effect of a more general model

To verify the generalization and evaluate the computation time of the proposed algorithm, the system to be identified adopts the Back and Tsoi NARMA model in [41,49], which is $W_{O}=\frac{0.0154+0.0462z^{-1}+0.0462z^{-2}+0.0154z^{-3}}{1-1.99z^{-1}+1.572z^{-2}-0.4583z^{-3}}$. For a time varying system, when iteration number smaller than 3000, the unknown coefficient vector is **W**_***O***_, when iteration number is larger than 3000, the unknown coefficient vector is 2**W**_***O***_. The system noise contains Gaussian white noise with SNR = 10dB. Parameters are set as the VSS-LHCAF {*μ* = 0.0005}, VSS-Llncosh {*α*_0_ = 0.1,h = 0.1,λ_0_ = 5,*μ* = 0.0005}, adaptive filter with secant cost {λ_0_ = 0.1,*μ* = 0.0005}, with arctangent cost {*μ* = 0.0005}, and the proposed HSF and IHSF algorithms {*μ* = 0.0005}.

## 5. Conclusions

In the context of system identification using the hyperbolic-type function algorithm under Gaussian white noise and impulsive interference, most of them have few parameters that need to be set, thereby increasing the complexity considerably. Besides, the adaptive estimation accuracy and convergence performance can be improved further; the hyperbolic sine function has not been discussed. So, in this paper, a family of the hyperbolic sine function is proposed, including a joint hyperbolic sine function (HSF) and inverse hyperbolic sine function (IHSF) as the cost function. Theoretically, it proves that both of the cost functions of HSF and IHSF are strongly convex at estimation error, and the computational complexity of HSF or IHSF is relatively low. Moreover, multi-types of experimental simulations show that compared with the VSS-LHCAF, VSS-Llncosh, adaptive filter with secant cost, with arctangent cost algorithms, the proposed algorithms are more robust Gaussian noise, impulsive interference, and various types of input signals. In short, that is, our methods could achieve a significantly lower steady-state error theoretically (i.e., MSE equal to SNR) and a faster convergence rate than prior methods (the VSS-LHCAF [42], VSS-Llncosh [45], adaptive filter with secant cost [46], with arctangent cost [48] algorithms) under different scenarios involving both uncorrelated and correlated input. Both theoretical analysis and simulations provided corroborated results. Although the HSF and IHSF algorithms have superior performance, the environment in actual engineering applications is complex nonlinear [50] and time-varying [51]and needs to be adjusted accordingly for different application scenarios.

## Supporting information

S1 File Code. (ZIP)
